# Musings on a quarter of a century in pediatric cardiac technology

**DOI:** 10.4103/0974-2069.58316

**Published:** 2009

**Authors:** Roger Restall

**Affiliations:** Royal Children’s Hospital, Melbourne, Australia

It has been a challenge and a privilege to run the diagnostic side of the cardiology department of the Royal Children’s Hospital (RCH) in Melbourne for the last 25 years. I joined the department as Chief Cardiac Technologist in September 1983, before the invention of CDs and two years before Vodafone introduced the first commercial mobile phone! Since that time there have been dramatic changes not only in the technology we work with, but also in the profession of cardiac technology.

I was the first technologist in the department with a university degree. ‘Cardiac Technicians,’ as they were then designated, usually came straight from school, between the ages of 16 and18 years. In 1985, I was part of a working group set up to form a professional body, the Association of Cardiac Technologists in Victoria (A.C.T.I.V.), for cardiac technologists in our state, and to create a career structure and a workplace award. Now a degree is a prerequisite for becoming a cardiac technologist, and a structured career pathway and pay structure is in place. In 2003, the Federal Government introduced a legislation requiring all sonographers to be accredited.

The department I joined in 1983, consisted of 13 people. We had three full-time and one sessional cardiologist, one trainee Fellow, three secretaries, and four technologists, including myself. Dr. Alex Venables was the director. Our department now numbers 40, with six full-time and one sessional cardiologist, six Fellows, nine administrative staff, a transplant coordinator, six nurses, and eleven technologists. Dr. Dan Penny is the present director.

In 1983, we performed 5,500 tests (2,870 ECGs, 450 Cardiac Catheters, 1,850 Echos, 185 Holters, 95 Exercise tests, and 50 Pacemaker checks). In 2008, we performed 18,000 tests (7,015 ECGs, 180 Cardiac Catheters, 8,870 Echos, 1,025 Holters, 510 Exercise tests, and 415 Pacemaker checks).

Those are the statistics, and they speak for themselves, but what of the experience? Over the years I have had the pleasure and privilege of working with many outstanding and dedicated colleagues. One of the greatest joys has been associating with more than 125 trainee Fellows who have passed through the department in the past 25 years. They have come from every corner of the globe to enhance their specialist careers and in return have enriched us with insights into their many different cultures. They are always bright, enthusiastic, and hard working despite finding themselves in a new cultural environment, and often with limited knowledge of English language. Over the years I have made some good friends and it is always a pleasure to catch up with them at conferences or on return visits. Many are now Heads of their own departments, and they are all making significant contributions in their home countries and abroad. Four of our current cardiologists, including the director, have also been Fellows in this department during my time.

Undoubtedly the most dramatic changes in cardiac technology over the past 25 years have been due to the introduction of computers and the speed with which the hardware and software have evolved in the fields of imaging, signal acquisition, and online analysis. In 1983, we were using single channel electrocardiogram (ECG) machines, and the tracings were then cut out and stuck onto special sheets. Holters were scanned on a screen with no printouts or algorithms. Pacemakers were in their infancy. We had one ATL 2-D Echo machine, which did not even have a spectral Doppler (color was still long in the future!) and an old Smith-Kline m-Mode Echo machine with a small round cathode-ray oscilloscope (CRO) screen mounted in a framework. The tracings came out on heat-sensitive paper and the measurements were taken off the paper with callipers. Dr. T. H. Goh was the lead cardiologist in Echo and had developed great expertise in m-Mode and early 2-D acquisition and interpretation. On occasion he was able to diagnose complex congenital conditions such as Transposition of the Great Arteries and Truncus Arteriosus from m-Mode tracings. It was to be many years, however, before the surgeons would accept 2-D Echo diagnosis as a basis for surgery, and cardiac catheterization remained the primary diagnostic tool well into the 1990s.

In 1988, Alex Venables retired and Dr. Jim Wilkinson, from Liverpool, England, was appointed as the new Director. Dr. Wilkinson immediately increased our Echo ‘fleet’ with the purchase of two ACUSON 128 × P10s and an HP SONOS 100 to go with our HP SONOS 1000 and HP SONOS 500. Prior to Dr. Wilkinson’s arrival the department had carried out peripheral clinics in Victoria and Tasmania. With the purchase of the (semi-) portable SONOS 100, Dr. Wilkinson expanded these peripatetic clinics throughout the state of Victoria and interstate in Tasmania. The cardiologists could now take the SONOS 100 and a technologist, to perform echos on location. This not only saved the inconvenience and expense of patients travelling to Melbourne for their consultations and tests, but also provided a closer link with the local pediatricians. We now carry out 70 peripheral clinic days a year.

Another of Dr. Wilkinson’s lasting initiatives was the heart transplant program. A transplant coordinator was appointed and the first transplant was carried out by Mr. Roger Mee, in October 1988. This first transplant patient, who was 15 at the time, is now married with a family and still very well and active after 21 years. Since then nearly 100 transplants have been performed.

Before Dr. Wilkinson arrived there were no computers in the department. Computer literate from the outset, Dr. Wilkinson soon introduced computers for word processing, and in 1989 / 1990, in conjunction with a local software company, a cardiac database program was developed using DOS. This is how the Cardiobase was born. Cardiobase has evolved over the years to a Windows-based version and is now available in a web version and used throughout Australasia and the UK. The department now has an extensive database of all diagnoses, surgeries, tests, and consultations covering nearly 20 years from 1 July, 1990. In recent years there has been a greater move toward digitized electronic storage of test results and patient information. Gone are the days of video tapes, cine films, and reams of paper traces. In 2008, we started using the Philips Xcelera system to digitally store all echos, cardiac angiography, and MRIs. In mid-2009 the GE MUSE system was introduced for the storage and reporting of ECGs. These modalities are downloaded directly using wireless technology or through local datapoints. Interfaces have been developed to provide the seamless transfer of information between the Hospital Information System (HIS), Cardiobase, Xcelera, and MUSE. This allows the instantaneous viewing of current studies at any one of the many workstations, and archived studies take only a few seconds to retrieve. In the past, with the storage on cine film, video tapes, and paper tracings it could take many minutes to review the current studies, and archived studies could take days to retrieve.

In 2001, Jim Wilkinson stepped down as Director and Dr. Dan Penny from Great Ormond Street Children’s Hospital in London was appointed to succeed him. Dr. Penny brought a new emphasis on both clinical and laboratory research to the department. His experience and drive to promote research culminated in the opening of a whole research floor in the new research wing of the hospital. This wonderfully designed area includes a number of units for clinical research as well as a small animal facility.

As methods in pediatric cardiology have grown more complex and specialized, Dr. Penny has now appointed specialist cardiologists to head the different disciplines within the department. Dr. Michael Cheung is Head of Cardiac Imaging, which includes Echo and MRI. Dr. Andrew Davis is Head of Arrhythmia and Electrophysiology (EP). Dr. Geoff Lane is Head of Intervention and Catheter Theater. Dr. Robert Weintraub is Head of Transplantation and Cardiomyopathy. Dr. Penny as well as being Director is Head of Research and Pulmonary Hypertension.

We now have 12 echo machines, 4 Philips iE 33s and 4 SONOS 5500s, a GE Vivid 7 and a Vivid i, a Siemens ×300, and an ACUSON Cypress portable machine. We have 3-D / 4-D transthoracic imaging with both pediatric and adult probes on one of our iE 33 machines. and five transesophageal echocardiography (TOE) probes including three pediatric multiplane and a 3-D probes. With the vastly improved image quality of our echo machines, together with a variety of applications available online, we are now able to provide more reliable diagnosis and our surgeons are now very comfortable operating on the echo evidence. Often in the 1980s and early 1990s very sick children would require general anesthesia and cardiac catheterization before undergoing complex surgery. Now a noninvasive transthoracic echo provides the same information.

In the arrhythmia area we have 5 GE MAC5500 / 5000 ECG machines. We use a Reynolds system for our Holter monitor analysis and have 30 Lifecard recorders, which will record up to 96 hours of three channel ECGs. In 1983, this amount of information would have required a number of audio cassettes, which was not practical to achieve. We also have a number of King of Hearts telemetric ECG recorders. We have approximately 200 patients with pacemakers and 40 with ICD devices. Our stress test system is Cambridge Heart, because of its ability to perform T-wave alternans analysis.

Our Catheter Theater has changed in functionality as well as technology over the last 25 years. In 1983, we had fixed, single plane fluoroscopy, and any oblique views had to be obtained by rotating the patient! Cine film was the recording medium for angiography and the ECG and hemodynamic tracings were recorded on photographic paper that was developed after the case, in the dark room! Recently, three generations later, we have just installed the latest Siemens biplane fluoroscopy equipment. In the 1980s our catheters were all diagnostic; in 2009, the majority of our catheters are interventional. These include myocardial biopsies, pulmonary and aortic valvuloplasties, atrial septal defect (ASD), ventricular septal defect (VSD), and patent ductus arteriosus (PDA) device closures, embolization, and stenting (mostly aortic and branch pulmonary artery). We recently carried out our first pulmonary valve replacement in a live transmission to the PICS meeting in Cairns, Australia. The introduction of these interventional procedures alleviates the necessity for bypass, open heart surgery, and lengthy hospital stays, not to mention large scars. Our new fluoroscopy equipment includes a Siemens X300 Echo machine for performing ICE (intracardiac echo) during interventional procedures. Over the years, visiting adult cardiologists have come to the RCH to perform EP studies. From late 2009, with the arrival of a new cardiologist, Andreas Pflaumer, and new EP equipment from GE, we started carrying out EP studies at the RCH.

What of the future? The current RCH was opened in the 1960s and is now struggling to keep abreast of modern practice and patient care. In 2006, the state government announced funding for a completely new Children’s Hospital. Work started in late 2007 with the new hospital due to open in the first half of 2011. Over the past 12 months I have been heavily involved in the design and fitting out of the diagnostic area of the new hospital. It will be an exciting time for all involved and opens up new possibilities and opportunities for the next generation. Who knows what an article like this will reveal in another 25 years time, and who will have the pleasure of writing it.

